# Thermal Processing of Watermeal for High-Protein Gluten-Free Rice Bread: Effects on Functional and Quality Properties

**DOI:** 10.3390/foods15203755

**Published:** 2026-10-21

**Authors:** Sunita Juaming, Clarica Clarica, Yuthana Phimolsiripol, Ravi Jadeja, Pavalee Chompoorat Tridtitanakiat

**Affiliations:** 1 Division of Product Development Technology, Faculty of Agro-Industry, Chiang Mai University, Chiang Mai 50100, Thailandclarica.c@student.i3l.ac.id (C.C.); yuthana.p@cmu.ac.th (Y.P.); 2 Department of Food Technology, Indonesia International Institute for Life Sciences, Jl. Pulomas Barat Kav. 88, Jakarta 13210, Indonesia; 3 Robert M. Kerr Food & Agricultural Products Center, Department of Animal and Food Sciences, Oklahoma State University, Stillwater, OK 74078, USA; 4 Lanna Rice Research Center, Chiang Mai University, Chiang Mai 50200, Thailand; 5 Sensory Evaluation and Consumer Testing Unit, Faculty of Agro-Industry, Chiang Mai University, Chiang Mai 50100, Thailand

**Keywords:** watermeal powder, gluten-free product development, functional nutrition, thermal processing

## Abstract

*Wolffia* spp., commonly known as watermeal, is a nutrient-rich aquatic plant with potential as a sustainable food ingredient; however, its high moisture content (approximately 95%) necessitates effective drying to preserve product quality. This study evaluated hot-air oven and vacuum drying at 60 and 70 °C and subsequently investigated the effects of incorporating 0–20% watermeal powder into gluten-free rice bread as a product innovation. Vacuum drying at 70 °C produced powder with the lowest moisture content and water activity and the highest protein content (26.44 g/100 g), total phenolic content, and solubility. Based on these combined physicochemical and nutritional characteristics, the vacuum-drying-at-70 °C powder was selected for incorporation into gluten-free rice bread. The powder contained all nine essential amino acids, although their concentrations varied among drying treatments. Watermeal powder incorporation increased the viscoelastic moduli of the batter and enhanced its structural resistance and recovery behavior. Incorporating 20% watermeal powder increased the protein and crude fiber contents of the bread from 5.65 to 8.15 g/100 g and from 1.33 to 5.08 g/100 g, respectively, while decreasing the specific volume from 2.64 to 1.83 cm^3^/g and loaf height from 43.92 to 39.17 mm. Bread staling was evaluated during 3 days of storage at 25 ± 2 °C. Despite these changes, the bread remained sensorially acceptable, with an overall-liking score of 6.0. These findings demonstrate the potential of dried watermeal as a nutrient-rich functional ingredient for gluten-free bread, providing nutritional enhancement while maintaining acceptable product quality.

## 1. Introduction

Increasing pressure on global food security has intensified interest in sustainable, nutrient-dense ingredients that deliver both high protein and dietary fiber. Watermeal (*Wolffia* spp.), an aquatic superfood and the smallest flowering plant on earth, fits this need due to its exceptionally high protein content and rapid, rootless free-floating growth, making it a promising alternative to conventional plant proteins for enhancing the nutritional quality of gluten-free bread. Watermeal, the smallest flowering plant on earth, is rootless and free-floating. Eleven species of *Wolffia* are recognized globally, with *Wolffia arrhiza* and *Wolffia globosa* commonly found in Thailand [1]. Notably, *W. globosa* offers potential benefits for prediabetic individuals, as it is rich in folic acid, iron, and vitamin B12. Its nutritional profile includes 20–45% protein, all essential amino acids, 1–7% fat, 10–43% carbohydrates, and 10–14.5% crude fiber [1,2]. To ensure safe consumption, *W. globosa* products require moisture <12% and water activity <0.6, emphasizing the need for an appropriate drying process. Taken together, the nutritional richness and sustainable growth characteristics of watermeal position it as a promising high-protein ingredient for improving gluten-free rice bread. Because of its naturally high moisture content, selecting an effective drying method is essential to producing a safe, stable powder suitable for food applications.

Drying plays a critical role in converting fresh watermeal into a stable powder suitable for food applications, as it not only lowers moisture and water activity but also influences the physicochemical integrity of the final product. The choice of drying method is particularly important because each technique, whether based on heated-air circulation or pressure-driven moisture removal, affects plant tissue components differently, including proteins, polysaccharides, and pigments [3]. In this study, hot-air oven drying and vacuum drying were selected for comparison due to their distinct thermal and mass-transfer mechanisms. Hot-air drying removes moisture through convective heat transfer, whereas vacuum drying reduces the boiling point and facilitates moisture migration under low pressure, allowing for gentler treatment of heat-sensitive compounds. Despite these advantages, incorporating watermeal into food products presents an additional challenge, as its natural green color and characteristic aquatic aroma may influence consumer acceptance, especially in gluten-free bread applications. Understanding how different drying methods affect the quality and sensory suitability of watermeal is therefore essential for its successful use as a sustainable high-protein ingredient.

Celiac disease and non-celiac gluten sensitivity require individuals to avoid gluten in order to prevent intestinal and systemic complications. Although the availability of gluten-free products has increased, many of these products still fall short nutritionally because they are often low in protein, fiber, and essential micronutrients [4,5]. This gap underscores the need for alternative ingredients that can improve both the nutritional value and sustainability of gluten-free foods. Watermeal has recently gained attention because of its unusually high protein and fiber content, yet its use in food applications is still limited. One of the main challenges is its naturally high moisture content, which means that drying is necessary before the powder can be incorporated into food products. Previous studies have primarily focused on drying optimization and the nutritional and bioactive properties of watermeal, while limited information is available on how different drying methods affect its subsequent functionality in food matrices. In particular, the relationship between the characteristics of dried watermeal, batter rheology, and the quality of gluten-free bread remains insufficiently understood. Therefore, the present study examined how hot-air oven drying and vacuum drying at two temperatures affect the physicochemical characteristics of watermeal powder [6]. The selected watermeal powder was subsequently evaluated for the technological functionality of the selected watermeal powder in gluten-free rice bread, including batter rheology, nutritional and physical properties, staling behavior, and sensory acceptability.

## 2. Materials and Methods

### 2.1. Preparation of Watermeal Powder

The initial watermeal samples were procured from the northern part of Thailand. A 100 g portion of fresh watermeal was placed in a baking dish and evenly spread to a thickness of 5 mm using a spatula within the dish (12 × 15 × 1.5 inches). The samples were then subjected to thermal treatments using two drying methods, namely hot-air oven drying (OD) and vacuum drying (VD), at two temperature levels (60 and 70 °C). The treatments were informed by a preliminary study. Thus, treatments used in this study were (1) OD 60 °C, (2) OD 70 °C, (3) VD 60 °C, and (4) VD 70 °C for 6 h with all treatments. The drying process was considered complete when the watermeal exhibited no visible surface moisture and reached a final moisture content below 10% based on visual and tactile assessment. Following the drying phase, the watermeal was ground into powder using a blender (Blendtec, Orem, UT, USA) and subsequently sieved through a 250 µm screen (60 US mesh) to obtain a uniform particle size. The ensuing watermeal powders were systematically stored in polyethylene bags, awaiting subsequent analytical procedures.

### 2.2. Gluten-Free Bread with Watermeal Powder Preparation

A commercial rice flour (Cho Heng Rice Vermicelli Factory Co., LTD. Bangkok, Thailand) with 6.9% protein, 1.3% fat, 0.5% total dietary fiber, and 79.8% carbohydrate by difference was applied in this study. Watermeal powder produced by vacuum drying at 70 °C (VD70) was selected for bread formulation based on its lowest moisture content and water activity and highest protein content, total phenolic content, and solubility among the evaluated drying treatment (Table 1). The gluten-free rice bread was prepared using these ingredients; rice flour 100%, xanthan gum 2.4%, instant dry yeast (1.9%), salt (1.6%), granulated sugar (15.6%), vegetable oil (11.8%), whole egg (32.4%), and water (60.3%). The amount of water was calculated based on the total flour weight. The watermeal powder with VD 70 °C was added at 0, 5, 10, 15, and 20% based on rice flour. The gluten-free rice bread was prepared following a modified method adapted from [7]. The gluten-free rice bread was prepared by mixing all dry ingredients (KitchenAid, St. Joseph, MI, USA) using a flat-beater attachment at low speed (60 rpm) for 1 min followed by the wet ingredients for 5 min at medium speed (111 rpm) until uniform. An amount of 200 g of batter sample was transferred to greased pans using spatula and proofed at 30 °C for 60 min in a convection oven (model EOG 601 X; AB Electrolux, Stockholm, Sweden). Baking was performed at 200 °C for 28 min, and the loaves were cooled before analysis for 60 min at ambient temperature and immediately transferred for analysis.

### 2.3. The Effect of Drying Method on Physicochemical Properties of Watermeal Powder

The content of moisture, protein, fat, ash, and fiber were measured following the AOAC guidelines from 2005 [8]. The calculation of total carbohydrate content was performed by subtracting the ash, lipid, protein, and fiber contents from 100. The results were expressed as g/100 g of sample on an as-is basis. The determination of water activity was performed using a water activity meter (AquaLab, Pullman, WA, USA) in standard sampling mode.

The assessment of solubility followed a modified procedure outlined by [9]. In this procedure, 0.2 g of the sample (*S*) was dissolved in 10 mL of deionized water over 10 min. Following dissolution, the solution was filtered through Whatman filter paper (*K*_1_) and then heated in an oven at 105 °C for 1 h. The filter paper, now containing the residue, was carefully weighed (*K*_2_). The solubility of the watermeal powder was then calculated using the formula indicated below:

(1) $$\mathrm{Solubility}\;(\%)=\frac{S-(K2-K1)}{s}\times 100$$

The water holding capacity (WHC) and oil holding capacity (OHC) were determined following the method reported by [10] with slight modifications. An amount of 0.5 g of the sample was dispersed in 20 mL of deionized water and shaken at 500 rpm for 30 min at room temperature. The suspension was centrifuged at 4000 rpm for 10 min. After centrifugation, the precipitate was collected and weighed. The WHC was expressed as grams of water retained per gram of sample (g/g). Additionally, pH levels were analyzed using a Mettler Toledo FiveEasy Plus pH meter FP20. The yield was determined as the percentage of the dried weight relative to the initial weight of the sample used.

The determination of total phenolic content followed the method outlined by [11] employing the Folin–Ciocalteu reagent. Initially, 10 g of samples underwent homogenization in 80% aqueous methanol at room temperature, facilitated by a shaker for a duration of 12 h. The resulting supernatant was separated by passing it through filter paper. Subsequently, A 20 μL aliquot of the sample was combined with 100 μL of 10% Folin-Ciocalteau reagent and 80 μL of 7.5% sodium carbonate. The sample was incubated for an additional 30 min in the dark place. The absorbance of the sample was measured at 765 nm using a Microplate reader TECAN. Gallic acid was employed as the reference standard for calibration, and the total phenolic content was reported as milligrams of gallic acid equivalents (GAE) per gram of sample.

The color of the samples was measured using a calibrated colorimeter (CR-400, Konica Minolta, Tokyo, Japan) after standard white calibration. This color assessment encompassed the evaluation of three primary indicators: L* (representing lightness), a* (indicating red intensity), and b* (reflecting yellow color intensity).

### 2.4. The Effect of Drying Method on Amino Acid Profile of Watermeal Powder

Amino acid profiling was performed based on the guidelines of Commission Directive 98/64/EC. Analyses were conducted using high-performance liquid chromatography (HPLC) following acid hydrolysis of the protein. For total amino acid determination, samples were hydrolyzed with 6 mol/L hydrochloric acid for 23 h. Prior to hydrolysis, cysteine and methionine were oxidized using performic acid/phenol to convert them into cysteic acid and methionine sulfone, respectively. Amino acids were separated by ion-exchange chromatography and quantified through reaction with ninhydrin, with photometric detection at 570 nm.

### 2.5. The Effect of Drying Method on FTIR Spectral Characteristics of Watermeal Powder

FTIR spectra of the samples were obtained using an FTIR-4700 type A spectrometer (Nicolet iS10, Thermo Fisher Scientific, Waltham, MA, USA). Spectra were recorded over 600–4000 cm^−1^ with a resolution of 4 cm^−1^. Each spectrum was collected from 32 accumulations using a TGS detector and a standard IR light source. The instrument was operated with cosine apodization, zero-filling on, and an automatic scanning speed (2 mm/s). The aperture size was set to 7.1 mm (auto mode), and an automatic 30,000 Hz filter was applied during data acquisition. The spectra were collected from 128 scans at a resolution of 4 cm^−1^ for each sample [12].

### 2.6. The Effect of Drying Method on Thermal Properties of Watermeal Powder

Thermogravimetric analysis (TGA) was performed by [13] with a few modifications to evaluate the thermal stability of the samples using a Thermogravimetric Analyzer (STARe System TGA/DSC 3+ Module -Auto Sample Robot, Mettler Toledo, Columbus, OH, USA). Approximately 10 mg of each sample was weighed into an aluminum crucible, and thermogravimetric data were recorded over a temperature range of 35 °C to 590 °C. The samples were heated at a constant rate of 10 °C·min^−1^ under a continuous nitrogen flow of 20 mL·min^−1^. Mass loss curves and derivative thermogravimetric (DTG) curves were obtained throughout the run to characterize thermal decomposition behavior.

### 2.7. Rheological Properties of Gluten-Free Rice Batter with Watermeal Powder

Rheological properties of batter samples without yeast addition were measured using a rotational rheometer HR30 (TA Instruments, New Castle, DE, USA). The frequency sweep test was performed to investigate the viscoelastic behavior of the sample over a range of frequencies. The strain level applied during the test was selected from the linear viscoelastic region (LVE), as determined by the amplitude sweep, typically between 0.1 and 0.5% strain. The frequency was varied from 0.1 to 10 Hz at a constant strain within the LVE to ensure that the sample structure remained undisturbed during measurement. This test allowed for the evaluation of the storage modulus (G′) and loss modulus (G″) as functions of frequency. The creep–recovery test was conducted to evaluate the structural stability and recovery behavior of the batter sample. Measurements were performed at 25 °C using a rheometer equipped with a 20 mm stainless steel parallel plate geometry (Plate SST ST 20 mm), with an upper plate diameter of 20 mm and a gap setting of 1 mm. The batter edges were trimmed with a spatula and covered to prevent structural changes from drying. The batter sample, which had a random initial shape due to subsequent flattening by the measuring system, was allowed to rest for 3 min before analysis to eliminate residual stresses caused by loading. During the creep phase, a constant shear stress of 20 Pa within the sample’s linear viscoelastic region was applied for 100 s. This was followed by a recovery phase in which the applied stress was reduced to 0 Pa for an additional 100 s to allow the batter structure to recover after deformation. The total duration of the measurement was 200 s [14].

### 2.8. Chemical Characteristics of Gluten-Free Rice Bread with Watermeal Powder

Proximate composition of the gluten-free rice breads containing watermeal powder was analyzed following the AACC Approved Methods for moisture (44-31.01) [8], protein (46-30.01) [15], fat (46-30-25.01) [16], ash (08-01.01) [17], and crude fiber (46-32-05.01) [14]. In addition, the total phenolic content of the samples was determined using the Folin–Ciocalteu method and expressed as milligrams of gallic acid equivalents per 100 g of sample [13].

### 2.9. Physical Characteristics of Gluten-Free Rice Bread with Watermeal Powder

The gluten-free rice breads containing five different levels of watermeal powder were prepared as described in Section 2.2. Water activity was measured as described in Section 2.3. The loaf volume and height were determined using manual measurements. Loaf height was recorded at the highest point of each bread sample using a caliper (mm) [17]. Bread volume was measured using the millet seed displacement method [16]. For calibration, rapeseed seeds were poured into a baking tray until filled. The baked bread was then placed in a beaker and filled with the calibrated millet seeds. The displaced millet was subsequently transferred into a measuring cylinder to determine the bread volume (cm^3^). The specific volume of the bread was calculated using the formula provided in Equation (2).

(2) $$\mathrm{Specific}\;\mathrm{Volume}\;({\mathrm{cm}}^{3}/g)=\frac{\mathrm{Loaf}\;\mathrm{volume}\;({\mathrm{cm}}^{3})}{\mathrm{Loaf}\;\mathrm{weight}\;(g)}$$

Crust and crumb color was tested as described in Section 2.3. Texture Profile Analysis (TPA) was conducted using a texture analyzer (TA-XT2, Texture Technologies Corp., Hamilton, MA, USA) according to [15]. Bread slices (2 cm thick) were analyzed within 20 min after cooling to room temperature using a 36 mm cylindrical probe at 40% compression and 1 mm/s test speed. Ten replicates were evaluated.

### 2.10. Staling Rate of Gluten-Free Rice Bread with Watermeal Powder

The staling behavior of gluten-free rice bread supplemented with watermeal powder was evaluated by monitoring changes in crumb texture during storage using texture profile analysis (TPA). Bread samples were packaged and stored under ambient conditions at room temperature (25 ± 2 °C) and analyzed at predetermined storage intervals. Crumb hardness was determined using a texture analyzer (TA-XT2, Texture Technologies Corp., USA) with a double-compression test. The increase in crumb hardness during storage was used as an indicator of bread staling, and the staling rate was determined based on changes in hardness over the storage period.

### 2.11. Sensory Evaluation of Gluten-Free Rice Bread with Watermeal Powder

A sensory evaluation was conducted using a 9-point hedonic scale with 50 panelists aged 21–35 years from the Faculty of Agro-Industry, Chiang Mai University. All participants provided written informed consent prior to the sensory evaluation. Participants were informed that their responses would be kept confidential, that participation was voluntary, and that they could withdraw from the evaluation at any time without providing a reason. Each participant indicated agreement by selecting the agreement checkbox and signing the informed consent form before tasting the samples. Each panelist evaluated coded samples of the control gluten-free rice bread (0%) and breads containing watermeal at 5, 10, 15, and 20 percent. Each sample was coded with a randomly assigned three-digit number, and the samples were presented to the participants in a randomized order. The attributes assessed included color, flavor, texture, and overall liking. Water was provided for palate cleansing, and all evaluations were performed in accordance with standard sensory laboratory procedures.

### 2.12. Statistical Analysis

The experiment was conducted using a completely randomized design (CRD). Statistical analyses were performed in SPSS software (Version 25, IBM Corp., Armonk, NY, USA). Analysis of variance (ANOVA) was applied to evaluate treatment effects, and mean differences were determined using Tukey’s multiple comparison test at a significance level of *p* < 0.05. Principal component analysis (PCA) was also conducted in SPSS to explore multivariate relationships among physicochemical and sensory attributes.

## 3. Results and Discussion

### 3.1. Physicochemical Properties of Watermeal Powder

Table 1 presented moisture and water activity of watermeal powder under different drying conditions. Among all treatments, vacuum drying at 70 °C produced the lowest moisture content (5.39 ± 0.17%) and the lowest water activity (0.40 ± 0.03). These reductions were likely due to the combined effects of elevated temperature and reduced pressure, which enhanced moisture migration from the interior to the surface of the material [18]. As moisture content decreased, water activity also declined because less free water remained available to support microbial growth or chemical reactions. The reduction in water activity was associated with a longer microbial lag phase and a reduced growth rate, thereby improving microbial stability and overall product safety [19]. Overall, both moisture content and water activity served as key indicators of product dryness and storage stability. The results indicated that vacuum drying, particularly at 70 °C, was the most effective condition for minimizing available water in watermeal powder, supporting improved shelf-life potential and suitability for subsequent food applications.

Proteins play diverse roles, serving as structural support, biochemical catalysts, hormones, enzymes, building blocks, and initiators of cellular death. There was a significant difference (*p* < 0.05) in protein content of watermeal powder, as presented in (Table 1), with vacuum drying at 70 °C (26.44 ± 0.04 g/100 g) displaying the highest protein content, followed by vacuum drying at 60 °C (25.12 ± 0.12 g/100 g), oven drying at 70 °C (24.80 ± 0.29 g/100 g), and oven drying at 60 °C (24.30 ± 0.12 g/100 g). The higher protein content observed at 70 °C in both oven and vacuum drying processes might be attributed to lower moisture content, causing a higher proportion of protein in the remaining matter and leading to an increase of protein content in the solid state [20].

The fat content of watermeal powder had a significant difference (*p* < 0.05) (Table 1) between each treatment, highlighting the highest fat content in the vacuum drying treatment at 70 °C (3.58 ± 1.07 g/100 g), followed by oven drying at 70 °C (3.46 ± 0.24 g/100 g), vacuum drying at 60 °C (3.31 ± 0.02 g/100 g), and oven drying at 60 °C (3.10 ± 0.09 g/100 g). Recent research delineated the nutritional composition of *Wolffia*, underscoring its notable phytosterol content at 50 mg/g, surpassing the 1–10 mg/g range found in many plant oils [2]. Consequently, the rising fat content observed in treatments at 70 °C could be ascribed to thermal processing, leading to the breakdown of oil cells and an augmented dissolution of oil in watermeal.

Ash content is an important indicator of powder quality and mineral richness [2,21]. Significant differences were observed among treatments (*p* < 0.05) (Table 1). Vacuum drying at 70 °C yielded the highest ash content, followed by vacuum drying at 60 °C, oven drying at 70 °C, and oven drying at 60 °C. The slight variations were likely due to minor mineral concentration changes during heating. Higher ash content in the 70 °C treatments reflected greater moisture loss, which concentrates mineral residues without degrading them.

As stated by [22], crude fiber pertained to the residue from plant foods after extraction with dilute acid and subsequent treatment with dilute alkali. The inclusion of fiber in foods served to modify their consistency, texture, rheological behavior, and sensory characteristics in the final products watermeal was recognized for its high crude fiber content, ranging from 10 to 14.5 g/100 g in dry weight. The crude fiber content of watermeal powder was presented in Table 1, with no significant differences (*p* > 0.05). The highest crude fiber content was exhibited in vacuum drying at 60 °C (13.67 ± 0.40 g/100 g), followed by oven drying at 60 °C (13.08 ± 0.21 g/100 g), vacuum drying at 70 °C (12.98 ± 0.35 g/100 g), and oven drying at 70 °C (12.76 ± 0.30 g/100 g) (Table 1).

The results indicated that vacuum drying and oven drying at 60 °C yielded higher crude fiber content compared to the temperature at 70 °C. This observation may be attributed to the higher drying temperature, leading to a lower crude fiber content, possibly due to the degradation of pectin or other fibers such as cellulose or hemicelluloses during the drying process [23]. Moreover, the extended duration of heating led to structural damage to crude fiber as continuous heat application is utilized, resulting in a lower crude fiber content in oven drying compared to vacuum drying.

Carbohydrates serve multiple functions, acting as an energy source, thickener, sweetener, and filler. Accurate analysis of carbohydrates is crucial for providing precise compositional information. As indicated in Table 1, the watermeal powders have significant differences (*p* < 0.05) among each treatment. The highest carbohydrate content was observed in oven drying at 60 °C (42.34 ± 0.14 g/100 g), followed by oven drying at 70 °C (41.78 ± 0.27 g/100 g), vacuum drying at 60 °C (40.52 ± 0.35 g/100 g), and vacuum drying at 70 °C (39.11 ± 0.20 g/100 g). The reduction in carbohydrate values observed in vacuum drying at 60 °C and 70 °C can be ascribed to the brief exposure of the sample to higher drying rate, thereby mitigating losses in certain nutrient components. The vacuum drying mechanism expedited the drying process, leading to decreased time requirements and consequently lower carbohydrate content [24]. Moreover, the concurrent loss of other components during oven drying treatments contributes to an apparent increase in the carbohydrate content of the dried samples.

The solubility results of watermeal powder are presented in Table 1. The vacuum-dried sample at 70 °C exhibited the highest solubility (49.71 ± 1.23%), which was significantly higher than all other treatments. The increased solubility observed under vacuum drying may be attributed to the greater reduction in particle size, improved particle uniformity, and the possible formation of more amorphous structures during drying, all of which enhance water interaction and dissolution [25]. Given its markedly higher solubility, the vacuum-dried watermeal powder at 70 °C is particularly suitable for incorporation into bakery systems, where improved dispersion and hydration are essential for achieving desirable dough and bread qualities.

WHC (19.02–19.84 g/g) and OHC (16.77–17.67 g/g) were statistically unaffected by the drying method (Table 1). This suggested that unlike solubility, these two functional properties were relatively robust to the temperature and drying mechanism variations tested here, likely because WHC and OHC were governed more by the overall structural composition than by the surface-level conformational changes that more strongly affect solubility.

The pH of fresh *Wolffia globosa* was reported to be approximately 5 [26], and its pH might increase as the plant matures. In this study, the drying treatments resulted in significant differences in pH values (*p* < 0.05). The highest pH was observed in the vacuum-dried sample at 60 °C (6.43 ± 0.01), followed by oven drying at 70 °C (6.32 ± 0.01), oven drying at 60 °C (6.30 ± 0.02), and vacuum drying at 70 °C (6.18 ± 0.00). The differences in pH among treatments may be attributed to the effects of temperature and drying time on organic acid volatilization. Higher temperatures can accelerate the loss of volatile organic acids, thereby reducing acidity and increasing the final pH of the dried material [27].

The drying method and temperature exerted a measurable influence on both the extraction yield and the total phenolic content of watermeal powder (Table 1). The yield values remained within a narrow range (4.52–4.89%), with VD70 producing the highest yield, followed by VD60, whereas OD60 and OD70 exhibited slightly lower yields. This pattern suggested that vacuum drying, particularly at elevated temperature, might enhance solid recovery efficiency, possibly due to reduced thermal degradation and improved moisture removal [28].

Total phenolic content (TPC) varied more distinctly across treatments. VD70 showed the highest TPC, followed by OD70, indicating that vacuum drying preserved phenolic compounds more effectively than oven drying. Both samples from oven and vacuum drying exposed to 60 °C yielded lower TPC values (Table 1). Exposure to heat and oxygen during hot-air drying could lead to the degradation of phenolic compounds through oxidation and thermal decomposition, resulting in lower total phenolic content compared with samples dried under vacuum conditions [22].

Table 1 presented clear differences in the color attributes of watermeal powder across drying methods. Oven drying at 60 °C produced the lightest green shade (highest L* value), whereas vacuum drying at 70 °C yielded the darkest appearance (lowest L* value). These changes were mainly associated with chlorophyll degradation, as elevated temperatures accelerate the conversion of chlorophyll to pheophytin, resulting in a darker green tone [29]. In addition, the more intense drying achieved under vacuum at 70 °C likely concentrated the remaining pigments, further contributing to the darker color. Overall, both the drying method and temperature played a decisive role in determining the visual quality of the resulting watermeal powder.

**Table 1:** Effect of thermal drying treatment on physicochemical properties of watermeal powder *.

Watermeal Powder Properties	Treatment			
	OD60	OD70	VD60	VD70
Moisture (%)	8.57 ± 0.26 ^a^	8.43 ± 0.21 ^a^	6.33 ± 1.29 ^b^	5.39 ± 0.17 ^c^
Protein (g/100 g)	24.30 ± 0.12 ^c^	24.80 ± 0.29 ^b^	25.12 ± 0.12 ^b^	26.44 ± 0.04 ^a^
Fat (g/100 g)	3.10 ± 0.09 ^b^	3.46 ± 0.24 ^a^	3.31 ± 0.02 ^ab^	3.58 ± 1.07 ^a^
Ash (g/100 g)	17.16 ± 0.04 ^b^	17.18 ± 0.05 ^b^	17.36 ± 0.10 ^b^	17.87 ± 0.11 ^a^
Crude Fiber (g/100 g)	13.08 ± 0.2 ^ab^	12.76 ± 0.3 ^b^	13.67 ± 0.4 ^a^	12.98 ± 0.3 ^b^
Carbohydrate (g/100 g)	42.34 ± 0.14 ^a^	41.78 ± 0.27 ^a^	40.52 ± 0.35 ^a^	39.11 ± 0.87 ^b^
Water activity	0.51 ± 0.00 ^a^	0.42 ± 0.00 ^b^	0.41 ± 0.00 ^c^	0.40 ± 0.00 ^c^
Solubility (%)	33.95 ± 0.84 ^d^	37.98 ± 0.38 ^c^	43.96 ± 1.74 ^b^	49.71 ± 1.23 ^a^
Water holding capacity (WHC) (g/g) ^ns^	19.84 ± 0.52	19.02 ± 0.07	19.51 ± 0.35	19.12 ± 0.60
Oil holding capacity (OHC) (g/g) ^ns^	17.67 ± 0.42	16.77 ± 1.65	17.22 ± 1.82	16.81 ± 0.02
pH	6.30 ± 0.02 ^b^	6.32 ± 0.01 ^b^	6.43 ± 0.01 ^a^	6.18 ± 0.00 ^c^
Yield (%)	4.83 ^b^	4.82 ^b^	4.52 ^c^	4.89 ^a^
Total phenolic content (mg Gallic acid/100 g)	13.17 ± 1.23 ^c^	14.64 ± 1.58 ^b^	12.50 ± 1.50 ^d^	15.06 ± 1.28 ^a^
L*	42.23 ± 0.96 ^a^	41.24 ± 0.67 ^ab^	40.42 ± 0.17 ^b^	37.69 ± 0.28 ^c^
a*	−10.45 ± 0.16 ^d^	−7.76 ± 0.18 ^c^	−6.91 ± 0.18 ^a^	−7.27 ± 0.09 ^b^
b*	24.36 ± 0.34 ^a^	22.08 ± 0.38 ^c^	23.08 ± 0.31 ^b^	24.66 ± 0.28 ^a^

* ^a–d^ Values are the average of triplicates ± standard deviation. Different superscripts in each column show significant statistical difference (*p* < 0.05), Tukey test. Thermal drying treatment (OD 60): hot-air oven drying 60 °C, (OD 70): hot-air oven drying 70 °C, (VD 60): vacuum drying 60 °C, and (VD 70): vacuum drying 70 °C. Reported as is basis, ^ns^: not statistically significant (*p* > 0.05).

### 3.2. Amino Acid Profile of Watermeal Powder

Table 2 also showed the results demonstrate that the drying method exerted a clear influence on the amino acid composition of watermeal powder. Vacuum drying at 60 °C produced the highest total amino acid content as well as the greatest proportion of essential amino acids, indicating more effective preservation of protein constituents under reduced oxygen and moderate heat [30]. In comparison, oven drying and vacuum drying at 70 °C showed lower values, which may be attributed to thermal degradation or oxidation of heat-labile amino acids. Overall, vacuum drying at 60 °C appears to be the most suitable method for maintaining amino acid integrity among the treatments evaluated. Although vacuum drying at 60 °C showed better preservation of total amino acids, vacuum drying at 70 °C was selected for bread application based on its overall combination of lower moisture content and water activity and higher protein content, total phenolic content, and solubility.

### 3.3. FTIR Spectral Characteristics of Watermeal Powder

The FT-IR spectra of watermeal powder subjected to different drying treatments are presented in Table 3. The broad band around 3423 cm^−1^ was assigned to O–H stretching associated with hydroxyl groups and hydrogen-bonded water, while the band at 1638–1655 cm^−1^ corresponded to the Amide I region, predominantly associated with C=O stretching of the protein backbone. The band around 1024 cm^−1^ was assigned to C–O stretching of polysaccharide structure. The major peak associated with the saccharide backbone (C–O stretching, 1024 cm^−1^) showed no pronounced shift across the control, OD, and VD samples at both 60 °C and 70 °C, suggesting that the overall polysaccharide structure was largely maintained after drying. Similarly, no pronounced peak shift was observed in the Amide I region (1638–1655 cm^−1^) for OD or VD compared with the control, suggesting that the applied drying conditions did not induce major changes in protein-related structures detectable by FTIR [31]. Among the heat-dried samples, the VD70 sample exhibited the lowest absorbance in the broad O–H stretching region around 3423 cm^−1^. This lower intensity may reflect lower residual water content and changes in hydrogen-bonding interactions following drying.

### 3.4. Thermal Properties of Watermeal Powder

**Figure 1:**
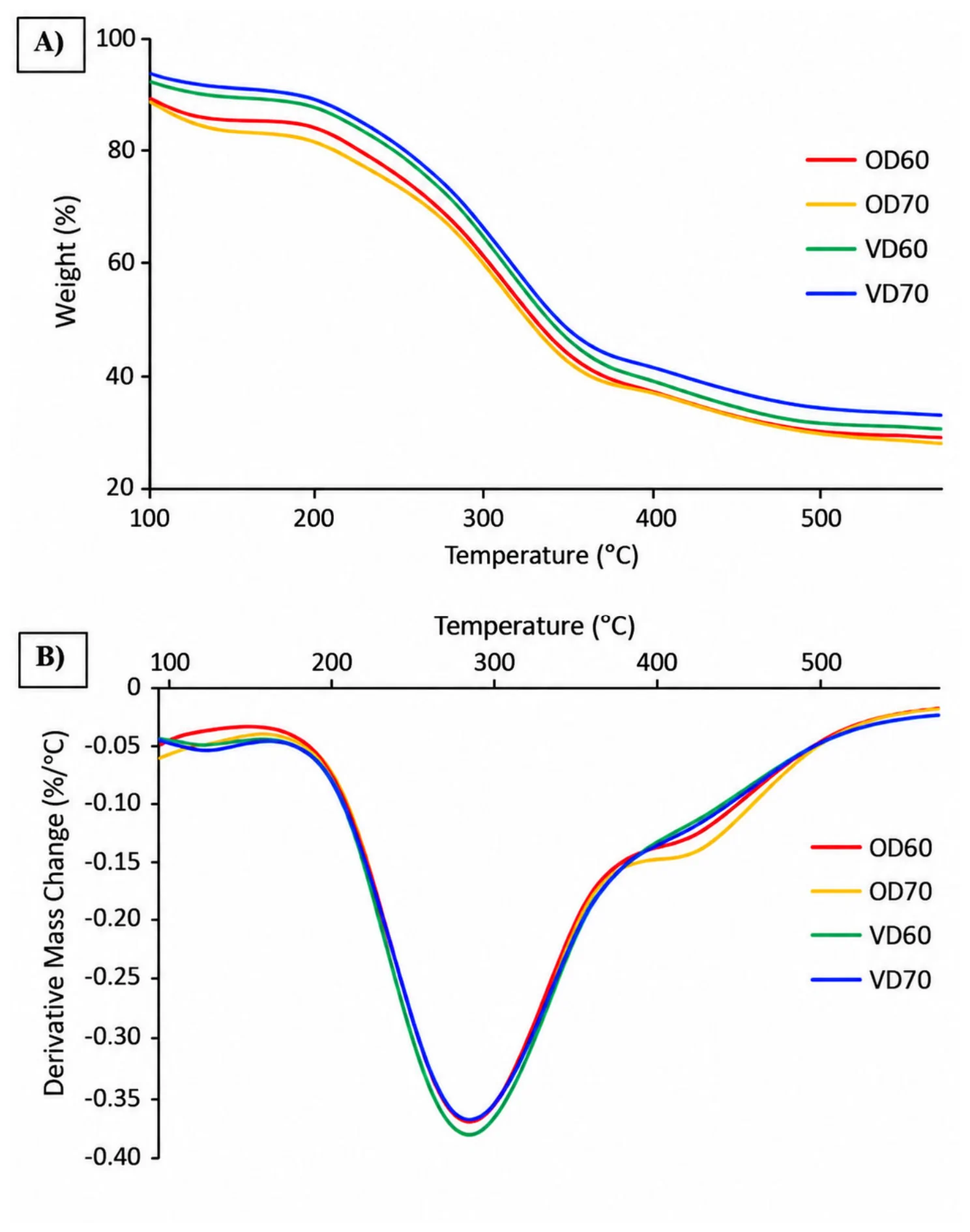
(**A**) Thermal gravimetric analysis (TGA). (**B**) Derivative thermogravimetric (DTG) curves of watermeal powder samples. with thermal drying treatment. (OD 60): hot-air oven drying 60 °C, (OD 70): hot-air oven drying 70 °C, (VD 60): vacuum drying 60 °C and (VD 70): vacuum drying 70 °C.

Thermal behavior of *Wolffia globosa* powders produced by oven and vacuum drying at 60 and 70 °C was evaluated using TGA and DTG analyses (Figure 1). All samples exhibited similar multi-stage decomposition patterns typical of protein- and polysaccharide-rich plant materials. Initial mass loss below 120 °C was attributed to the removal of residual moisture [21]. Vacuum-dried powders, particularly at 60 °C, showed lower mass loss in this region, indicating improved moisture stability due to reduced boiling temperature and limited oxygen exposure. The main degradation stage occurred between 200 and 350 °C and was associated with the thermal decomposition of proteins and soluble carbohydrates [32]. Oven-dried samples, especially at 70 °C, exhibited earlier degradation onset and higher DTG peak intensities, suggesting greater thermal and oxidative sensitivity. In contrast, vacuum-dried powders showed delayed decomposition and lower maximum weight-loss rates, reflecting better preservation of macromolecular structures. At temperatures above 400 °C, all samples underwent further degradation related to thermally stable structural components, with vacuum-dried powders retaining slightly higher residual mass, indicative of a denser and more thermally stable matrix [33].

### 3.5. Rheological Properties of Gluten-Free Rice Batter with Watermeal Powder

**Figure 2:**
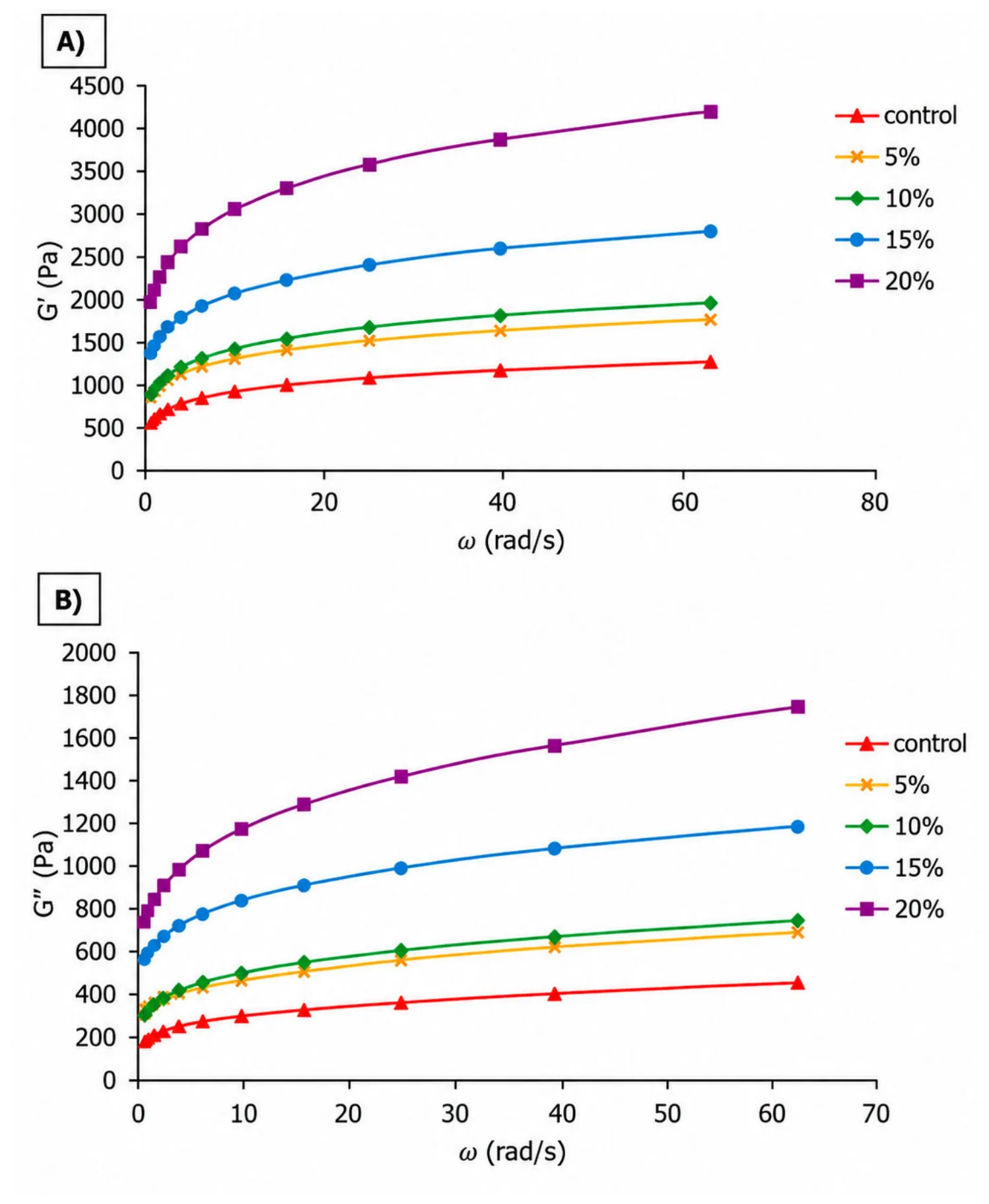
(**A**) The storage modulus (G′). (**B**) The loss modulus (G″) as a function of frequency (*ω*) of gluten-free batter with watermeal powder incorporation at different levels.

Figure 1 shows the frequency sweep results demonstrated that all batter formulations exhibited typical weak gel-like behavior, characterized by storage modulus (G′) values higher than loss modulus (G″) across the tested frequency range (0.1–10 Hz). As shown in Figure 2, both G′ and G″ increased with increasing frequency, indicating a frequency-dependent viscoelastic response. The dominance of G′ over G″ suggests that the batter systems possessed predominantly elastic characteristics, which is consistent with structured, semi-solid food systems. Incorporation of watermeal powder resulted in noticeable changes in the magnitude of both moduli, reflecting modifications in the internal network structure of the gluten-free batter [34,35]. The increases in G′ and G″ with watermeal powder incorporation may be attributed to the higher protein and fiber contents introduced by watermeal powder, which enhanced water binding and promoted interactions among batter components, resulting in a more structured viscoelastic network. However, the stronger batter network did not necessarily improve bread expansion, as the high water-binding capacity of fiber and the absence of gluten-forming proteins may have restricted gas-cell expansion and retention during proofing and baking. This behavior is consistent with the observed decreases in specific volume and loaf height at higher watermeal powder incorporation levels.

**Figure 3:**
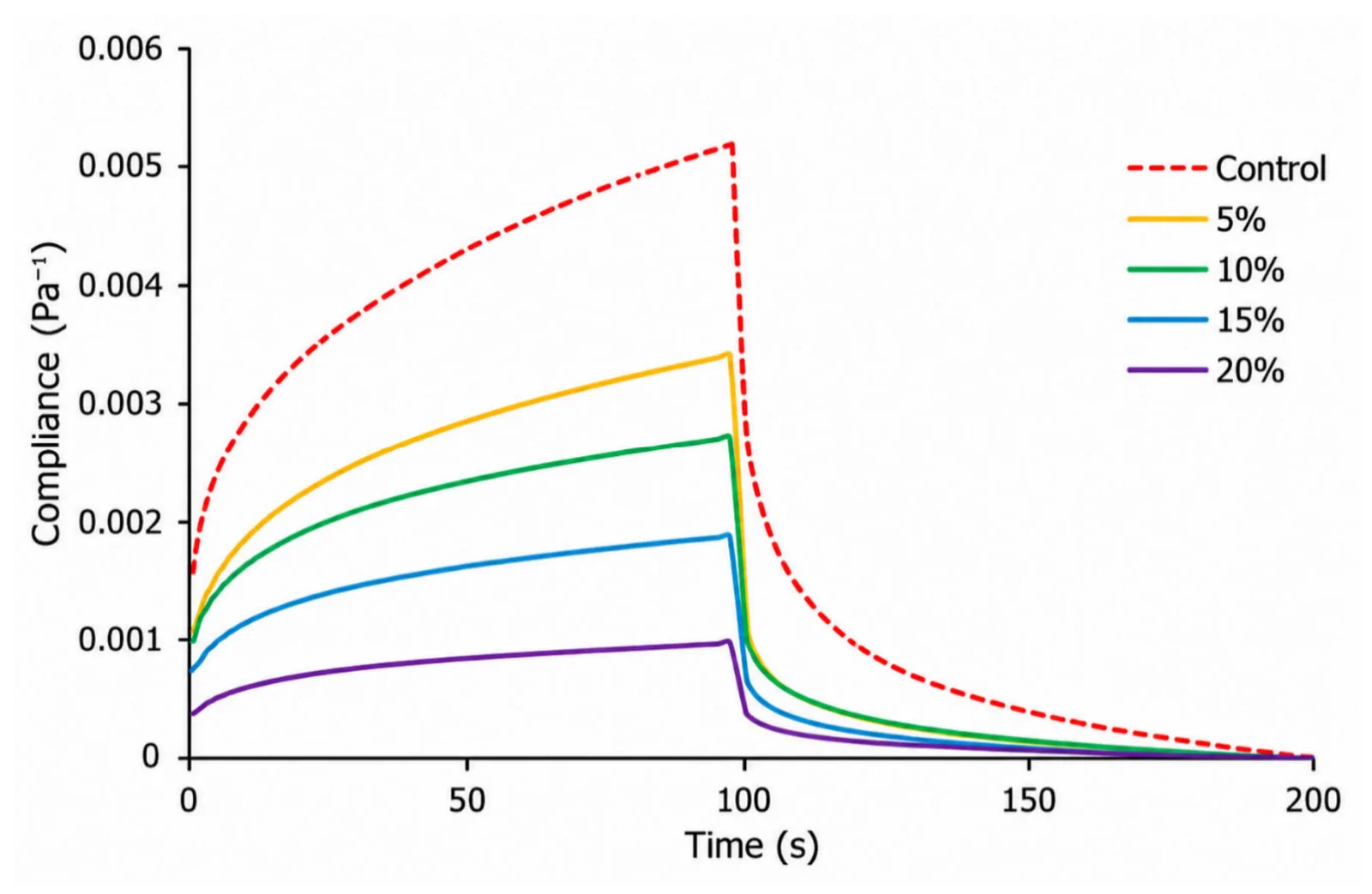
The compliance of gluten-free batter with watermeal powder incorporation at different levels as a function of time.

The creep–recovery test further confirmed the structural characteristics of the batter systems. When a constant stress (20 Pa) within the linear viscoelastic region was applied, all samples exhibited time-dependent deformation during the creep phase, followed by partial recovery once the stress was removed. The compliance curves (Figure 3) indicated that watermeal-enriched batters showed reduced maximum deformation compared to the control, suggesting enhanced structural resistance to applied stress. During the recovery phase, the samples demonstrated partial elastic recovery, indicating the presence of a viscoelastic network rather than purely viscous flow behavior [36]. The decrease in total compliance and improved recovery in watermeal-containing samples suggest stronger internal interactions and improved structural stability.

### 3.6. Chemical Characteristics of Gluten-Free Rice Bread with Watermeal Powder

Table 4 showed moisture content significantly decreased with increasing watermeal powder concentration, remaining within the acceptable range of 35–45% for bread freshness and stability. This reduction was attributed to the high fiber content (10–14.5%) and hemicellulose (29%) in watermeal’s cell wall, which exhibited strong hygroscopic and hydrophilic properties that absorbed water from the bread matrix [37].

Protein content significantly increased with higher watermeal powder incorporation, attributed to the high protein content (20–45% in fresh state) of *Wolffia.* (Table 4), Although drying processes reduced this content through protein dissolution. Protein played a crucial role in water retention and starch gel stabilization during gelatinization, while also contributing to swelling and emulsifying properties [38].

Fat content increased significantly with watermeal powder incorporation due to the naturally occurring fat content of watermeal (1–7%) (Table 4). Lipids influenced starch gelatinization through the formation of starch–lipid complexes, particularly between amylose and fatty acid chains, which can modify gelatinization behavior and crumb structure. However, at higher fat levels, the use of liquid oils in the formulation reduced gas retention efficiency compared with solid fats, thereby limiting dough expansion during baking and resulting in a lower bread volume [39].

Ash content increased proportionally with watermeal powder concentration, rising from the typical gluten-free rice bread range of 1.11–1.34% due to the 6–7% ash content present in watermeal powder, indicating enhanced mineral content that contributes to nutritional value assessment and food quality evaluation (Table 4).

Crude fiber content increased significantly with higher watermeal powder incorporation, reflecting the naturally high crude fiber content of *Wolffia globosa* (10–14.5%) (Table 4). At lower incorporation levels, crude fiber contributed positively to water retention and improved textural properties by enhancing moisture distribution within the dough matrix. However, at higher concentrations, the high water absorption capacity of dietary fiber diluted structure-forming components and interfered with gas cell stabilization during baking, leading to reduced dough expansion and a denser bread structure [40].

Table 4 further showed carbohydrate content significantly decreased with increasing watermeal powder concentrations, falling below the typical gluten-free rice bread range of 33.32–55.92%, primarily due to the dilution effect of other nutritional constituents present in watermeal powder and the calculation methodology employed. This reduction was further attributed to the decreased proportion of high-carbohydrate flours (rice flour: 87.7–92.5%, tapioca flour: 83.17%, arrowroot flour: 70.25%) in the bread formulation, as they were partially replaced by watermeal powder, which contains lower carbohydrate content and higher proportions of protein, fiber, and other nutrients [2].

The total phenolic content (TPC) of gluten-free rice bread exhibited a significant upward trend (*p* < 0.05) (Table 4). in correlation with the dosage of VD70 watermeal powder. While the control sample recorded the lowest TPC at 262.86 mg GAE/100 g, supplementation at levels of 5%, 10%, 15%, and 20% progressively enhanced these values to 363.65, 411.16, 416.50, and 427.20 mg GAE/100 g, respectively. However, the TPC of gluten-free rice bread was slightly lower than that of VD70 watermeal powder alone (441.28 mg GAE/100 g), likely due to dilution and partial degradation or interactions of phenolic compounds during baking. Thermal processing can reduce extractable phenolics through oxidation [41,42,43,44].

### 3.7. Physical Characteristics of Gluten-Free Rice Bread with Watermeal Powder

Both specific volume and loaf height decreased significantly as watermeal powder concentration increased, attributed to low gas retention capacity, high protein content lacking gluten-forming proteins, and reduced moisture content. This decrease could lead to harder bread with more compact cells [45]. The gluten-free rice bread remained within acceptable ranges for specific volume (1.83–2.64 cm^3^/g) and loaf height (39.17–43.92 mm) as reported in previous studies, indicating that watermeal incorporation up to 20% still produced quality gluten-free rice bread products (Table 4).

The incorporation of watermeal powder significantly darkened both crust and crumb color, with lightness (L*) decreasing from 53.13 to 30.09 in crust and 69.99 to 34.04 in crumb as concentration increased from 0% to 20% (Table 4). This darkening was primarily caused by the Maillard reaction between amino acids from the high protein content of watermeal and reducing sugars, producing brown melanoidins [46]. The b* values also decreased due to the inherent dark green color of watermeal powder (L* 41.58, a* −7.87, b* 25.41), while a* values showed inconsistent patterns attributed to color degradation during the drying process.

Table 4 shows that hardness increased significantly with increasing watermeal powder concentration, as dietary fibers contributed to thickening of cell walls and the encapsulation of air bubbles, while the reduction in moisture content further enhanced bread rigidity [47]. Conversely, cohesiveness and springiness decreased with higher fiber levels, as fibers interfered with internal bonding forces within the dough matrix, reduced CO_2_ gas retention during baking, and interacted with the aqueous phase surrounding starch granules, thereby limiting starch hydration and compromising the overall structural integrity of the dough [6]. Further formulation optimization, particularly adjustment of water content and hydrocolloid levels, may help compensate for the high water-binding capacity of watermeal powder and improve batter expansion, gas retention, and crumb softness.

### 3.8. Staling Rate of Gluten-Free Rice Bread with Watermeal Powder

The staling rate of gluten-free rice bread, expressed as hardness, increased significantly (*p* < 0.05) over the three-day storage period for all formulations except the bread containing 20% watermeal powder, which showed no significant change in hardness from Day 0 to Day 3 (Table 5). The control bread (0%) exhibited the greatest increase in hardness, rising from 1.13 to 1.32 (an increase of approximately 17%), followed by the 10% formulation (1.17 to 1.34), the 5% formulation (1.16 to 1.29), and the 15% formulation (1.16 to 1.25). In contrast, the 20% formulation remained essentially unchanged throughout storage (1.18 to 1.16), indicating that the staling rate decreased progressively with increasing watermeal powder incorporation, with the 15% and 20% formulations showing the most pronounced anti-staling effect.

On Day 0, hardness values among the five formulations were not markedly different, indicating limited initial separation between samples. As storage progressed, differences among formulations became more pronounced. By Day 2 and Day 3, the bread containing 20% watermeal powder showed significantly lower hardness than the control and 5% and 10% formulations, while the 15% formulation occupied an intermediate position.

### 3.9. Sensory Evaluation of Gluten-Free Rice Bread with Watermeal Powder

**Figure 4:**
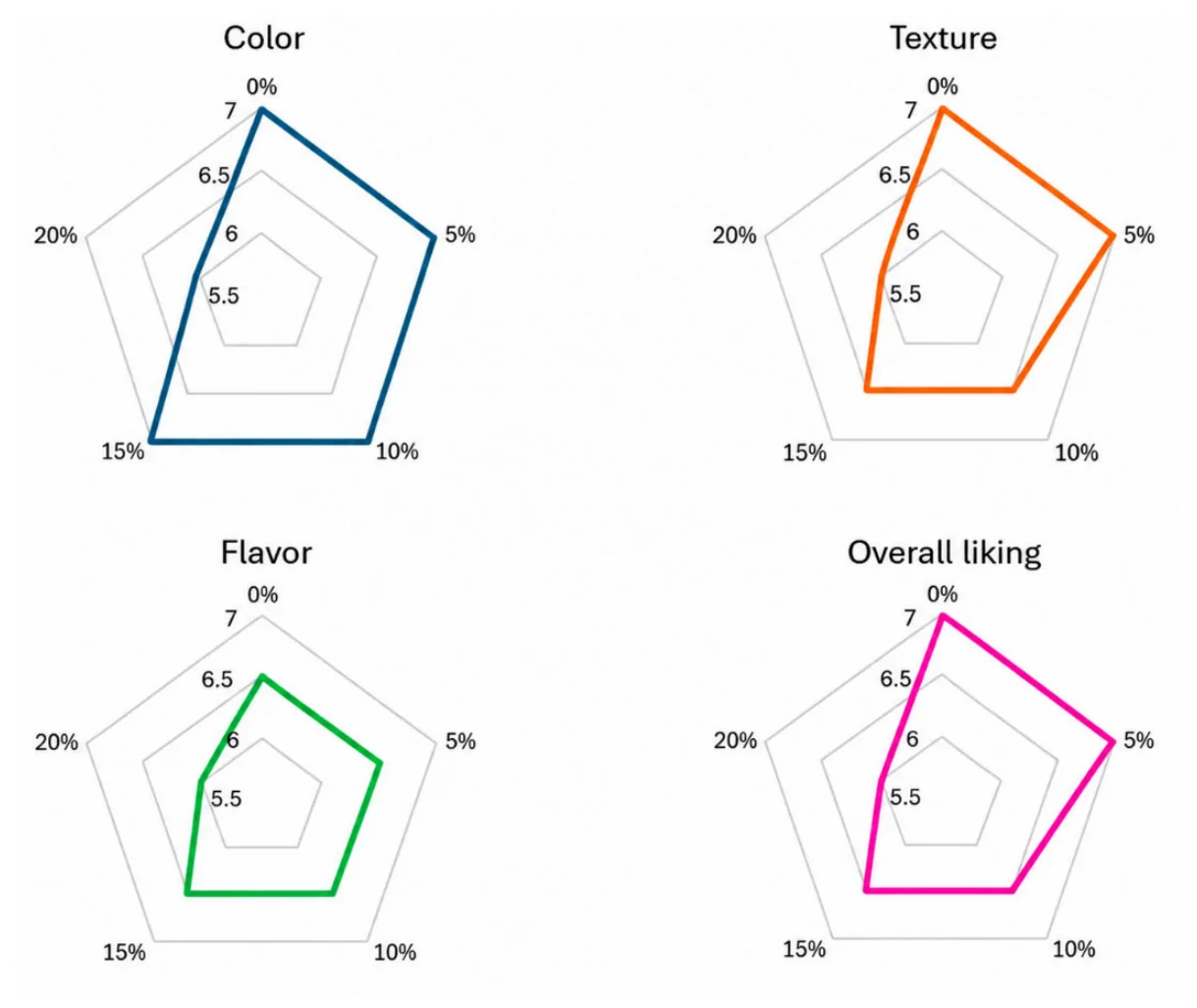
Spider diagram for sensory evaluation of gluten-free rice bread for various content of watermeal powder (0%, 5%, 10%, 15%, and 20% watermeal powder incorporation treated with vacuum drying at 70 °C).

**Figure 5:**
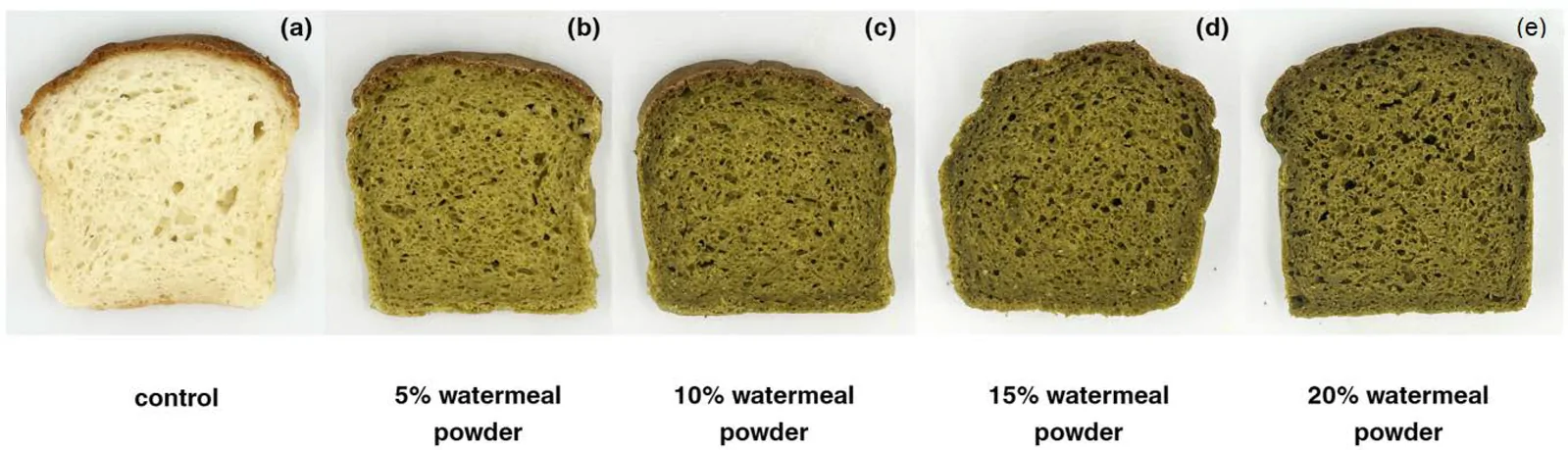
Representative loaves of slices produced by watermeal powder treated with vacuum drying at 70 °C. For various contents (0%, 5%, 10%, 15%, and 20% watermeal powder incorporation).

The sensory evaluation of gluten-free rice breads formulated with 0–20% incorporation of watermeal powder (Table 4 and Figure 4). Panelists assessed color, texture, flavor, and overall liking on a scale of approximately 6.0 to 7.0. The 10% substitution level consistently received the highest scores across all attributes, achieving ratings of 7.0 for color, texture, and overall liking. Low-to-moderate incorporation (5–10%) enhanced color and flavor, whereas higher levels (15–20%) reduced acceptability, likely due to the darker color and changes in texture (Figure 5). Overall, the lowest score recorded was around 6 for bread containing 20% watermeal powder, which still falls within an acceptable range for consumer acceptance.

### 3.10. Principal Component Analysis

**Figure 6:**
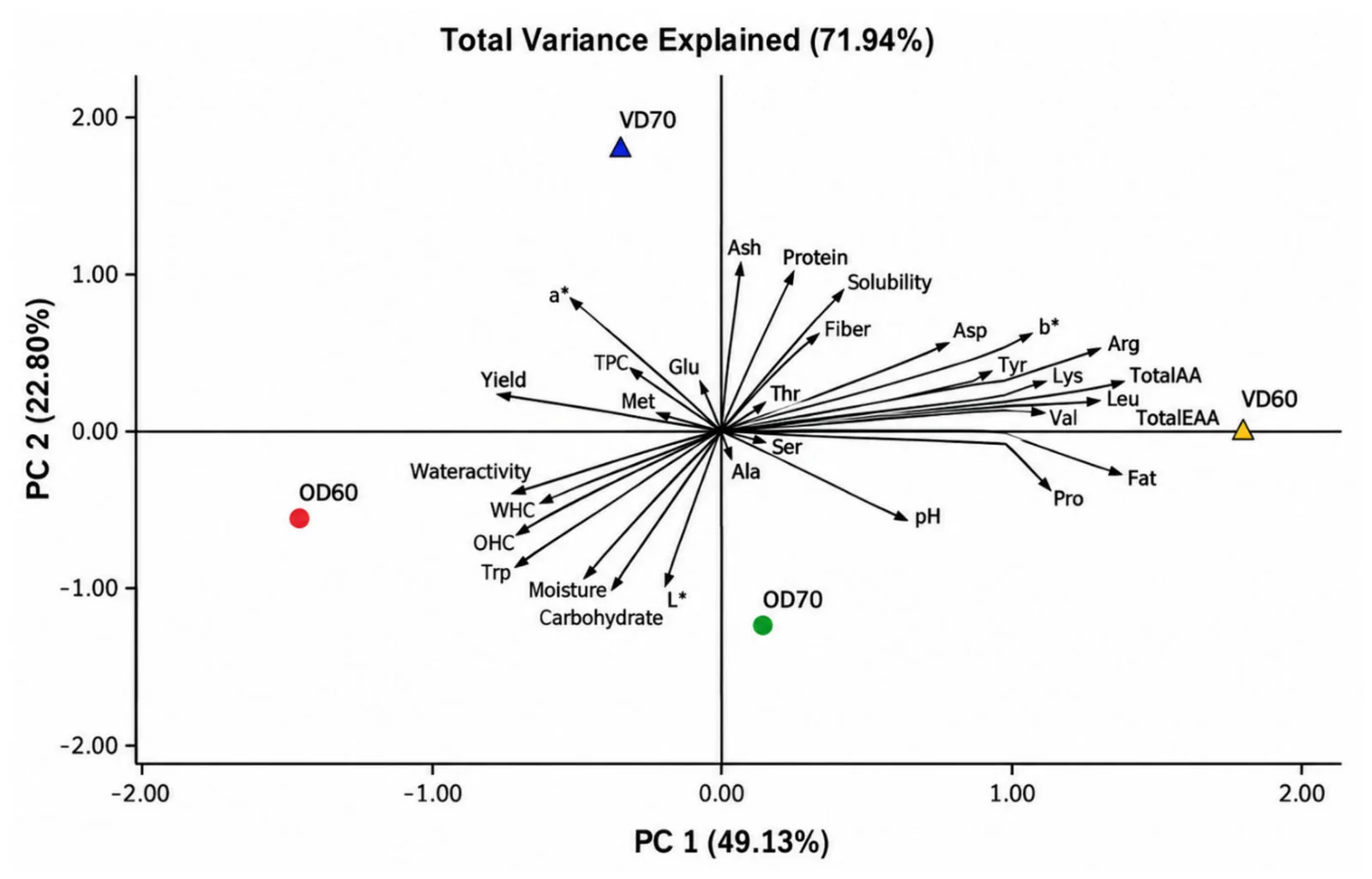
The biplot of the principal component analysis (PCA) based on the physicochemical propperties and amino acid profile of thermal treated watermeal powder. (OD 60): hot-air oven drying 60 °C, (OD 70): hot-air oven drying 70 °C, (VD 60): vacuum drying 60 °C, and (VD 70): vacuum drying 70 °C. TPC: Total phenolic acid, Asp: Asphatic acid, Thr: Threonine, Ser: Serine, Glu: Glutamic acid, Gly: Glycine, Ala: Alanine Val: Valine, Met: Metthionine, Ile: Isoleucine, Leu: Leucine, Tyr: Tyrosine, Phe: Phenylalanine, His: Histidine, Lys: Lysine, Arg: Arginine Pro: Proline, Trp: Tryptophan, TotalAA: total amino acid, TotalEAA: total essential amino acid.

Principal component analysis (PCA) was employed to illustrate the intricate relationships among physicochemical properties, amino acid composition, and the resultant quality attributes of bread derived from various formulas (Figure 6). In the raw-material PCA, PC1 and PC2 explained 49.13% and 22.80% of the variance, respectively. VD60 was positioned in the direction of total amino acids, total essential amino acids, and several individual amino acids, whereas VD70 was more closely associated with TPC, yield, and a*. OD60 was associated with moisture, water activity, WHC, OHC, and carbohydrate content. Furthermore, key nutritional variables, including protein, fat, and fiber content, were clustered together and showed a stronger association with the vacuum-dried samples compared to other formulas. This collective evidence suggests that the drying technique directly impacts the stability of nutritional components and the overall molecular structure of the raw material.

**Figure 7:**
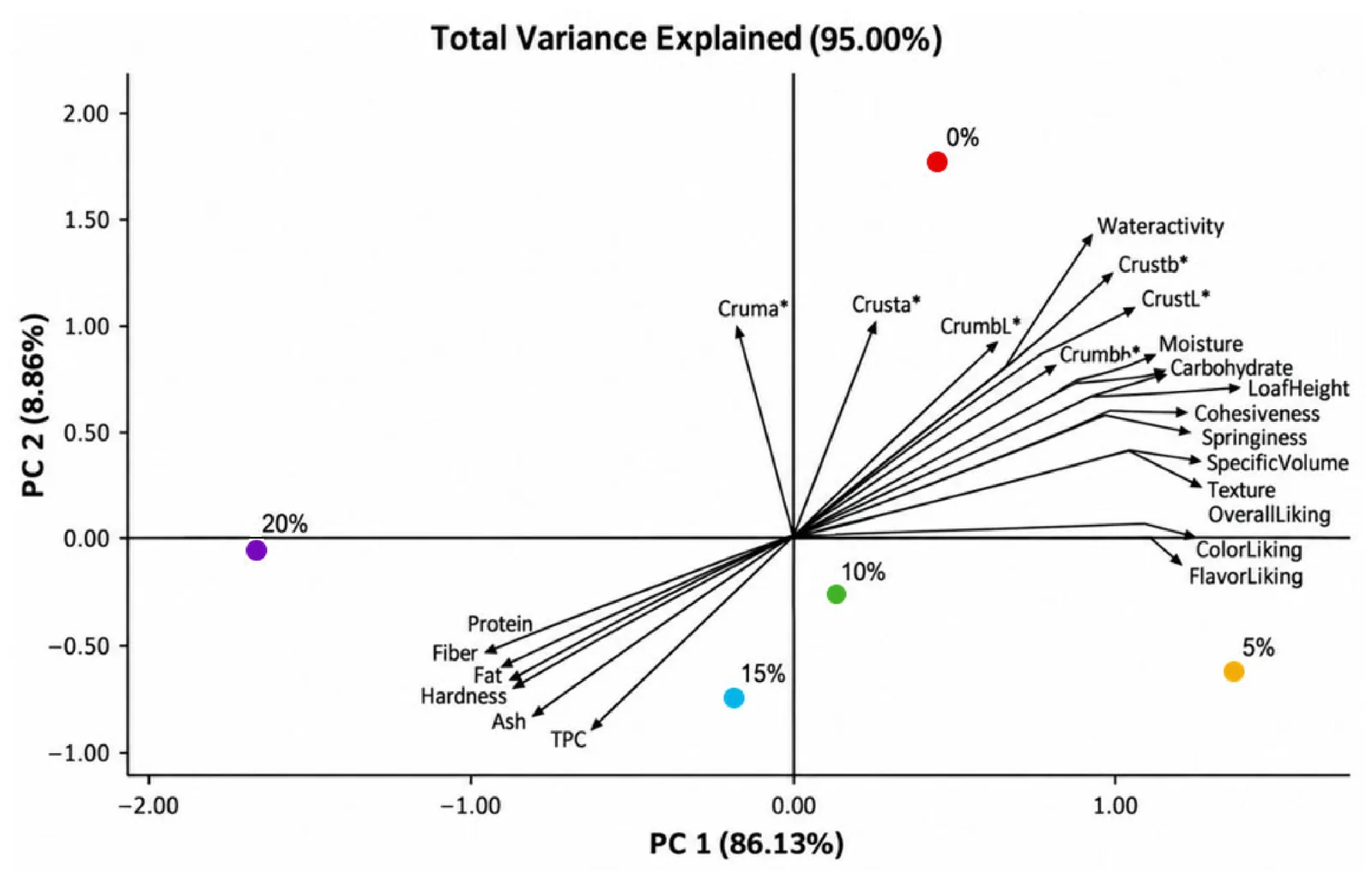
The biplot of the principal component analysis (PCA) based on the physicochemical properties of gluten-free rice bread for various content of watermeal powder treated with vacuum drying at 70 °C (0%, 5%, 10%, 15%, and 20% watermeal powder incorporation).

Compared with Prosridee et al. [6], who focused on aquaculture and hot-air drying optimization, the present study further evaluated vacuum drying and the technological functionality of dried watermeal in gluten-free rice bread, including batter rheology, bread quality, staling behavior, and sensory acceptability. The final bread quality data yielded a remarkably high explanatory power, with PC1 accounting for 86.13% and PC2 for 8.86% of the variance (Figure 7). The results positioning the samples indicated that formulas incorporating a low ingredient ratio (5%) were situated within the quadrant positively correlated with beneficial physical quality parameters of the bread crumb. These optimal attributes included specific volume, loaf height, springiness, and cohesiveness. However, as the ingredient incorporation level was increased to higher ratios (10–20%), these samples tended to correlate highly with protein; fat; fiber content; and, notably, hardness. This outcome demonstrates that increasing the quantity of the added ingredient contributes to a denser structure and a firmer texture in the finished bread product.

## 4. Conclusions

Vacuum drying at 70 °C produced watermeal powder with the lowest moisture content and water activity and the highest protein, fat, ash, solubility, and total phenolic content among the evaluated treatments. In contrast, vacuum drying at 60 °C produced the highest total amino acid content. Therefore, VD70 was selected for bread development based on its low moisture content and water activity and high protein content, total phenolic content, and solubility rather than because it was superior in every measured property. Watermeal powder incorporation enhanced batter viscoelasticity, as indicated by higher storage and loss moduli and lower compliance. Increasing the incorporation of VD70 watermeal powder enhanced the protein, fat, ash, crude fiber, and total phenolic contents of gluten-free rice bread but reduced specific volume and loaf height and increased hardness. Sensory scores remained within the acceptable range, although the 20% formulation received lower scores than formulations containing 0–15% watermeal powder. Overall, a 5–10% incorporation level provided a more favorable balance between nutritional enhancement, physical quality, and sensory acceptability. Further studies could address formulation optimization and extended shelf-life evaluation, including microbial stability and quality changes during prolonged storage.

## Figures and Tables

**Table 2:** Effect of thermal drying treatment on amino acid profile of watermeal powder *.

Amino Acid Profile	Treatment							
	OD60		OD70		VD60		VD70	
	mg/100 g	% Total	mg/100 g	% Total	mg/100 g	% Total	mg/100 g	% Total
Aspartic acid	2736.18 ^d^	13.25	2900.08 ^c^	13.03	2935.27 ^b^	12.12	2948.92 ^a^	13.13
Serine	965.15 ^d^	4.67	1050.31 ^b^	4.72	1135.01 ^a^	4.68	1036.9 ^c^	4.62
Glutamic Acid	2521.15 ^d^	12.21	2699.04 ^c^	12.12	3046.05 ^a^	12.57	2906.57 ^b^	12.94
Glycine	1134.09 ^c^	5.49	1234.62 ^b^	5.55	1340.05 ^a^	5.53	1234.2 ^b^	5.49
Alanine	2012.31 ^d^	9.74	2090.37 ^b^	9.39	2346.51 ^a^	9.69	2070.8 ^c^	9.22
Tyrosine	650.2 ^d^	3.15	704.94 ^c^	3.17	787.23 ^a^	3.25	722.48 ^b^	3.22
Arginine	1348.16 ^d^	6.53	1493.84 ^c^	6.71	1564.37 ^a^	6.46	1501.73 ^b^	6.68
Proline	954.15 ^d^	4.62	1039.32 ^b^	4.67	1073.55 ^a^	4.43	999.03 ^c^	4.45
Threonine *	947.04 ^d^	4.59	1021.85 ^b^	4.59	1099.69 ^a^	4.54	1015.31 ^c^	4.52
Valine *	1369.95 ^c^	6.63	1466.39 ^b^	6.59	1616.72 ^a^	6.67	1466.66 ^b^	6.53
Methionine *	248.83 ^d^	1.20	281.48 ^b^	1.26	310.07 ^a^	1.28	271.91 ^c^	1.21
Isoleucine *	828.95 ^c^	4.01	925.57 ^b^	4.16	1015.37 ^a^	4.19	925.44 ^b^	4.12
Leucine *	1778.03 ^d^	8.61	1890.3 ^b^	8.49	2032.33 ^a^	8.39	1887.69 ^c^	8.40
Phenylalanine *	1176.53 ^d^	5.70	1263.74 ^c^	5.68	1632.24 ^a^	6.74	1287.7 ^b^	5.73
Histidine *	450.69 ^c^	2.18	498.72 ^b^	2.24	529.48 ^a^	2.19	498.01 ^b^	2.22
Lysine *	1213.26 ^d^	5.88	1377.71 ^c^	6.19	1462.26 ^a^	6.04	1383.62 ^b^	6.16
Tryptophan *	316.3 ^b^	1.53	324.74 ^a^	1.46	300.37 ^d^	1.24	307.33 ^c^	1.37
**Total amino acid content**	20,650.97 ^d^	100.00	22,263.02 ^c^	100.00	24,226.57 ^a^	100.00	22,464.30 ^b^	100.00
**Total Essential amino acid**	9276.62 ^c^	44.92	11,571.65 ^a^	51.98	9998.53 ^b^	41.27	9043.67 ^d^	40.26

* ^a–d^ Values are followed by different letters within a row are significantly different at (*p* < 0.05). * Essential amino acid, Thermal drying treatment (OD 60): hot-air oven drying 60 °C, (OD 70): hot-air oven drying 70 °C, (VD 60): vacuum drying 60 °C, and (VD 70): vacuum drying 70 °C.

**Table 3:** FTIR spectrum of watermeal powder under different treatments.

			Treatment			
Frequency (cm^−1^)	Absorbing Feature	Compound Class	OD60	OD70	VD60	VD70
3600–3300	O - H stretching	Alcohol	3423	3422	3399	3423
2500–3000	C - H stretching	Alkane	2923	2923	2922	2924
1690	C=O stretching	Primary amide	1639	1655	1638	1638
1050–1085	C-O stretching	Primary alcohol	1035	1024	1036	1100

(OD 60): hot-air oven drying 60 °C, (OD 70): hot-air oven drying 70 °C, (VD 60): vacuum drying 60 °C and (VD 70): vacuum drying 70 °C.

**Table 4:** Effect of watermeal powder incorporation on properties of gluten-free rice bread *.

Gluten-Free Rice Bread Properties	Watermeal Powder Incorporation				
	0%	5%	10%	15%	20%
**Proximate composition**					
Moisture (g/100 g)	41.35 ± 0.05 ^a^	40.83 ± 0.10 ^b^	40.56 ± 0.08 ^b^	40.25 ± 0.15 ^b^	39.92 ± 0.11 ^c^
Protein (g/100 g)	5.65 ± 0.14 ^e^	6.01 ± 0.17 ^b^	6.71 ± 0.25 ^c^	7.49 ± 0.19 ^d^	8.15 ± 0.21 ^a^
Fat (g/100 g)	6.46 ± 0.13 ^e^	7.73 ± 0.08 ^d^	8.36 ± 0.17 ^c^	8.93 ± 0.08 ^b^	10.27 ± 0.19 ^a^
Ash (g/100 g)	1.52 ± 0.05 ^e^	2.78 ± 0.07 ^d^	3.22 ± 0.05 ^c^	3.68 ± 0.12 ^b^	4.25 ± 0.17 ^a^
Crude Fiber (g/100 g)	1.33 ± 0.14 ^e^	2.25 ± 0.12 ^d^	3.20 ± 0.09 ^c^	4.09 ± 0.14 ^b^	5.08 ± 0.20 ^a^
Carbohydrate (g/100 g)	43.60 ± 0.10 ^a^	40.39 ± 0.18 ^b^	37.94 ± 0.28 ^c^	35.53 ± 0.35 ^d^	32.30 ± 0.12 ^e^
Total phenolic content (mg GAE/100 g)	262.86 ^d^	363.65 ^c^	411.16 ^b^	416.50 ^b^	427.20 ^a^
**Physical characteristics**					
Water activity	0.99 ± 0.01 ^a^	0.97 ± 0.05 ^bc^	0.96 ± 0.01 ^ab^	0.96 ± 0.01 ^a^	0.95 ± 0.00 ^a^
Specific Volume (cm^3^/g)	2.64 ± 0.24 ^a^	2.54 ± 0.13 ^a^	2.26 ± 0.15 ^b^	1.96 ± 0.35 ^c^	1.83 ± 0.20 ^c^
Loaf Height (mm)	43.92 ± 0.21 ^a^	42.43 ± 0.10 ^b^	41.35 ± 0.19 ^c^	39.71 ± 0.27 ^d^	39.17 ± 0.32 ^d^
**Crust color**					
L*	53.13 ± 0.86 ^a^	43.16 ± 0.50 ^b^	35.37± 1.02 ^c^	32.47 ± 0.84 ^d^	30.09 ± 0.67 ^e^
a*	15.43 ± 0.79 ^a^	5.16 ± 1.14 ^d^	8.31 ± 0.71 ^b^	5.81 ± 0.74 ^c^	4.67 ± 0.41 ^e^
b*	53.13 ± 0.86 ^a^	43.16 ± 0.50 ^b^	35.37 ± 1.06 ^c^	32.47 ± 0.840 ^d^	30.09 ± 0.67 ^e^
**Crumb color**					
L*	69.99 ± 1.58 ^a^	51.06 ± 1.58 ^b^	42.27 ± 0.21 ^c^	41.05 ± 1.99 ^d^	34.04 ± 0.98 ^e^
a*	1.05± 0.44 ^a^	0.10 ± 0.03 ^b^	0.62 ± 0.15 ^ab^	0.49 ± 0.36 ^b^	0.50 ± 0.20 ^b^
b*	18.35 ± 0.21 ^a^	17.34 ± 0.03 ^b^	16.88 ± 0.16 ^c^	16.05 ± 0.56 ^d^	15.81 ± 0.55 ^e^
**Textural properties**					
Hardness (N)	3.16 ± 0.28 ^a^	4.33 ± 0.28 ^b^	4.50 ± 0.50 ^b^	5.16 ± 0.28 ^c^	6.16 ± 0.28 ^d^
Cohesiveness (N)	2.06 ± 0.05 ^e^	1.72 ± 0.05 ^d^	1.13 ± 0.01 ^c^	0.85 ± 0.11 ^b^	0.55 ± 0.07 ^a^
Springiness (N)	2.12 ± 0.02 ^e^	1.92 ± 0.05 ^d^	1.45 ± 0.11 ^c^	1.14 ± 0.04 ^b^	0.84 ± 0.04 ^a^
**Sensory characteristics**					
Color	7.0 ± 0.50 ^a^	7.0 ± 0.65 ^a^	7.0 ± 0.81 ^a^	7.0 ± 0.50 ^a^	6.0 ± 0.90 ^b^
Flavor	6.5 ± 0.70 ^a^	6.5 ± 0.50 ^a^	6.5 ± 0.62 ^a^	6.5 ± 0.85 ^a^	6.0 ± 0.51 ^b^
Texture	7.0 ± 0.43 ^a^	7.0 ± 1.00 ^a^	6.5 ± 1.02 ^a^	6.5 ± 0.75 ^a^	6.0 ± 0.50 ^b^
Overall-liking	7.0 ± 0.65 ^a^	7.0 ± 0.52 ^a^	6.5 ± 0.74 ^a^	6.5 ± 0.85 ^a^	6.0 ± 0.79 ^b^

* ^a–e^ Values are the average of triplicates ± standard deviation. Different superscripts in each column show significant statistical difference (*p* < 0.05), Tukey test. Thermal drying treatment (OD 60): hot-air oven drying 60 °C, (OD 70): hot-air oven drying 70 °C, (VD 60): vacuum drying 60 °C and (VD 70): vacuum drying 70 °C. Reported as-is basis.

**Table 5:** Staling rate of gluten-free rice bread during three days of storage.

Staling Rate	Watermeal Powder Incorporation				
	0%	5%	10%	15%	20%
**Day 0**	1.13 ± 0.01 ^bC^	1.16 ± 0.00 ^aB^	1.17 ± 0.00 ^aC^	1.16 ± 0.01 ^aB^	1.18 ± 0.02 ^aA^
**Day 1**	1.20 ± 0.05 ^abB^	1.17 ± 0.01 ^bB^	1.23 ± 0.03 ^aBC^	1.20 ± 0.01 ^abAB^	1.20 ± 0.02 ^abA^
**Day 2**	1.31 ± 0.1 ^aA^	1.23 ± 0.06 ^abA^	1.30 ± 0.07 ^aAB^	1.25 ± 0.05 ^abA^	1.18 ± 0.04 ^bA^
**Day 3**	1.32 ± 0.07 ^aA^	1.29 ± 0.1 ^aA^	1.34 ± 0.08 ^aA^	1.25 ± 0.1 ^abA^	1.16 ± 0.05 ^bA^

Different lowercase letters in the same line indicate significant differences between samples on the same day (*p* < 0.05); different uppercase letters in the same column indicate days’ significant differences for the same sample (*p* < 0.05), of gluten-free rice bread for various content (0%, 5%, 10%, 15%, and 20% watermeal powder incorporation) of watermeal powder treated with vacuum drying at 70 °C.

## Data Availability

The data presented in this study are available on request from the corresponding author.
